# Occlusion Robust Wheat Ear Counting Algorithm Based on Deep Learning

**DOI:** 10.3389/fpls.2021.645899

**Published:** 2021-06-11

**Authors:** Yiding Wang, Yuxin Qin, Jiali Cui

**Affiliations:** School of Information Science and Technology, North China University of Technology, Beijing, China

**Keywords:** wheat ear counting, transfer learning, image augmentation, attention module, deep learning

## Abstract

Counting the number of wheat ears in images under natural light is an important way to evaluate the crop yield, thus, it is of great significance to modern intelligent agriculture. However, the distribution of wheat ears is dense, so the occlusion and overlap problem appears in almost every wheat image. It is difficult for traditional image processing methods to solve occlusion problem due to the deficiency of high-level semantic features, while existing deep learning based counting methods did not solve the occlusion efficiently. This article proposes an improved EfficientDet-D0 object detection model for wheat ear counting, and focuses on solving occlusion. First, the transfer learning method is employed in the pre-training of the model backbone network to extract the high-level semantic features of wheat ears. Secondly, an image augmentation method Random-Cutout is proposed, in which some rectangles are selected and erased according to the number and size of the wheat ears in the images to simulate occlusion in real wheat images. Finally, convolutional block attention module (CBAM) is adopted into the EfficientDet-D0 model after the backbone, which makes the model refine the features, pay more attention to the wheat ears and suppress other useless background information. Extensive experiments are done by feeding the features to detection layer, showing that the counting accuracy of the improved EfficientDet-D0 model reaches 94%, which is about 2% higher than the original model, and false detection rate is 5.8%, which is the lowest among comparative methods.

## Introduction

The number of wheat ears is used as the essential information to study wheat yield (Prystupa et al., 2004; Peltonen-Sainio et al., 2007; Ferrante et al., 2017). Accurate monitoring of the number of wheat ears is necessary for growers to predict wheat harvest and growth trends. The counting of wheat ears is usually done manually, which is an extremely time-consuming work (Liu et al., 2016). In large-scale planting scenarios, the accuracy of manual counting will increase with the increase of the number of wheats. Therefore, it is indispensable to develop an efficient and automatic wheat ear counting method.

**FIGURE 1:**
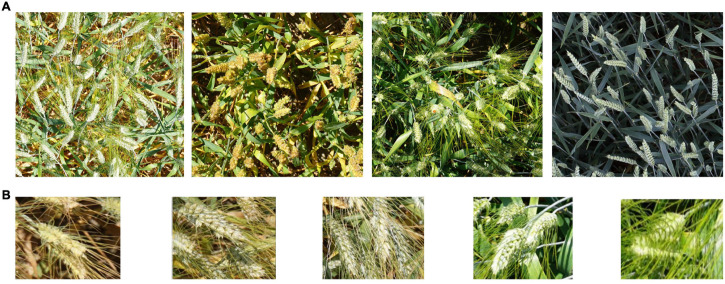
Wheat images in the Global Wheat dataset. **(A)** Different varieties, light, maturity **(B)** Examples for occlusion and overlap.

Traditionally, automatic counting methods based on image processing have been successfully used in practical applications, such as plant leaf counting and fruit counting (Giuffrida et al., 2015; Mussadiq et al., 2015; Maldonado and Barbosa, 2016; Stein et al., 2016; Aich and Stavness, 2017; Barré et al., 2017; Dobrescu et al., 2017). These methods fall into two categories. In the first class of conventional methods, the color of the target objects is extracted and set as positive samples. The background color is set as negative samples, and then traditional machine learning classification methods, such as Support Vector Machine (SVM) are used to separate the target and background in the images. But in the actual wheat ear counting task, the varieties and maturity of wheat will be different (Figure 1), which lies in the fact that the preset positive sample color cannot represent wheat ears under all conditions. Methods in the second category used threshold segmentation algorithms, such as Watershed Algorithm (Bleau and Leon, 2000). Although this type of method reduces the dependence on color information, the segmentation threshold is determined by experience, which makes the algorithm have no generalization ability and low robustness.

The previous wheat ear counting methods were mainly realized by manual counting and traditional image processing methods, which has great room for improvement in precision and generalization ability. In contrast, for counting complex background and dense object distribution, deep learning has inherent advantages that can overcome some of the shortcomings of traditional methods. There are two ways to implement deep learning based wheat ear counting algorithm: semantic segmentation and object detection. The process of counting using the semantic segmentation method is reproduced below. Above all, the ears of wheat are labeled pixel by pixel in the original images, and the regions containing the ears are positive samples and other regions are negative samples. After the image is annotated, the fully convolution network such as Unet (Ronneberger et al., 2015), FCN (Long et al., 2015), etc. is usually trained in way of encoder-decoder (Grbovic et al., 2019; Sadeghi-Tehran et al., 2019; Misra et al., 2020; Xu X. et al., 2020). The trained full convolutional network can segment each wheat ear in the input images and output it in the form of a mask. There are two difficulties with this approach. First, training the fully convolution network requires pixel-level annotation. The time cost of this annotation method is almost the same as that of manually counting the number of ears in the image. Second, the mask output by fully convolutional network is not directly related to the number of wheat ears. Solving this problem usually involves designing multifaceted post-processing steps. By using object detection implementation counting, these problems can be avoided effectively. In this way, people roughly mark the positions of the upper left and lower right corners of the ears, and the detection results can be directly converted to the number of ears. Hasan et al. (2018) adopted R-CNN (Girshick et al., 2014) and Madec et al. (2019) adopted the Faster-RCNN (Ren et al., 2017) method to calculate the number of wheat ears. Later, more researchers utilized object detection methods to model wheat ear counting tasks (Mohanty et al., 2016; Xiong et al., 2019; Lu and Cao, 2020). Therefore, wheat ear counting based on deep learning was realized by object detection methods, which makes the algorithm easy to be applied in practice.

With the rapid development of deep learning theory, object detection methods based on deep learning have become a new paradigm in machine learning in recent years. Compared with traditional image processing technologies, Convolutional Neural Networks (CNN) is invariant to geometric transformation, illumination, and background differences. This feature overcomes the deficiencies of many traditional technologies. Since the advent of the R-CNN network in 2014, deep learning has made rapid progress in object detection. Then YOLO (Redmon et al., 2016), SSD (Liu et al., 2016), R-FCN (Dai et al., 2016), etc. continuously refresh the object detection accuracy level. In 2019, Google launched the EfficientDet family of models and feature fusion module called BiFPN (Tan et al., 2020). EfficientDet achieves state-of-the-art accuracy with fewer parameters compared to the previous object detection and semantic segmentation model. It contains a total of eight versions from D0 to D7. The best results can always be achieved under the constraints of the computing resources of different devices. At the same time, BiFPN also shows the best efficiency in multi-scale feature fusion. At present, the deep learning model based on EfficientDet and BiFPN is being applied to a variety of research fields, such as forest fire prevention (Xu et al., 2021), estimation of fashion landmarks (Kim et al., 2021), detection of garbage scattering areas (You et al., 2020), etc.

However, deep learning technology is not a universal method, and there will be problems in wheat ear detection and counting tasks. The species of wheat, for example, differ from other plants in that individual wheat plants have multiple ears. Therefore, there will be dozens of wheat ears in an image, which will cause serious occlusion problems (Figure 1). Occlusion and overlap will cause acute deviations in the detection and counting results of the model. In the study of Hasan et al. (2018) and Madec et al. (2019), counting accurately reached 86 and 91%, respectively. However, it seems that the occlusion and overlap of wheat cannot be effectively solved.

In this study, wheat ear counting adopts object detection method. So, the main objective is aimed at improving the EfficientDet-D0 model. In detecting and counting wheat ears, it focuses on addressing the problems of occlusion and overlap in the wheat ear images.

## Materials and Methods

**FIGURE 2:**
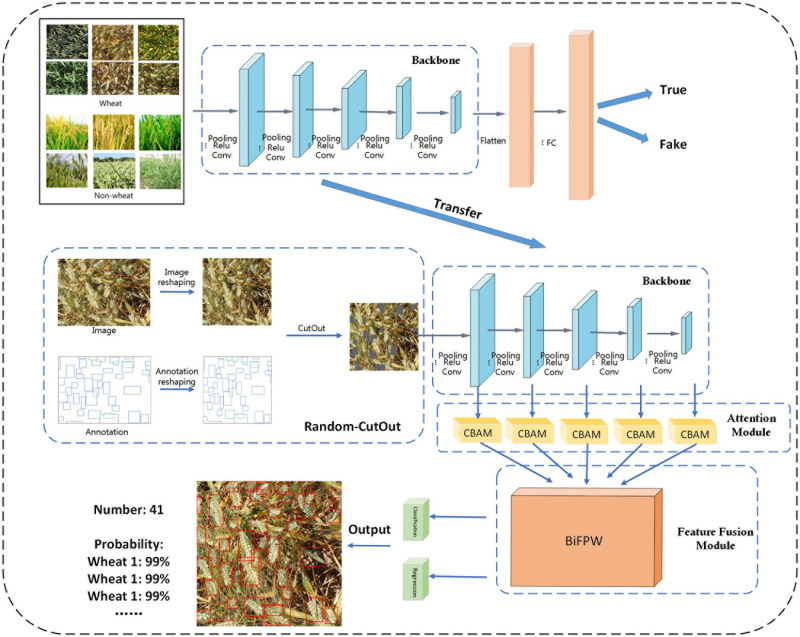
The proposed pipeline for robust counting and detection of wheat eras.

In this study, the pipeline of the wheat ear counting algorithm based on the EfficientDet-D0 model is shown in Figure 2. The pipeline comprises four important parts: transfer learning, Random-Cutout image augmentation, attention module, and feature fusion module. First, the backbone network of the Effcientdet-D0 is separately trained utilizing transfer learning. Then Random-Cutout is used to augment the input images. After that, the attention module will refine the feature map output by the backbone network. Finally, feature fusion module fuses feature maps with different resolution and semantic information, followed by detection layer and Non-Maximum Suppression (NMS) to obtain the final detection results.

### Dataset and Platform

The data used in this study are from the public data set called Global Wheat (David et al., 2020). Eight institutions lead the data set in seven countries: University of Tokyo, Arvalis, INRAE, University of Saskatchewan, ETH Zürich, University of Queensland, Nanjing Agricultural University, and Rothamsted Research. To better gauge the performance for unseen genotypes, environments, and observational conditions, this dataset covers multiple regions, including Europe (France, United Kingdom, Germany), North America (Canada), Asia (China, Japan), and Australia. All the 3,365 images were randomly split into training set, validation set and test set without overlap. 2,693 (∼80%) images were selected as the training set, 336 images (∼10%) were used as the validation set, and the remaining 336 images (∼10%) were used as the test set. The performance of the final model is all obtained on the test set, and the data in the test set will never participate in training.

In this work, all models are trained and tested on the same device, which consists of an Intel E5-2603 V4 CPU, 1TB hard disk, and two Titan X graphics cards. The operating environment is Ubuntu16.0.4, tensorflow2.3.0 and Python3.7.

### EfficientDet-D0 and BiFPN

The research in this article is based on DfficientDet-D0 object detection model. Its performance can surpass classic one-stage networks such as YOLOV3 and SSD, but its floating-point operations per second (FLPOS) is about 1/28 of homogeneous one-stage networks. Lightweight parameters enable DfficientDet-D0 to be easily deployed to hardware in practical applications, and the single inference time can satisfy the real-time counting work.

EfficientDet-D0 consists of two principal parts: the backbone network and the feature fusion module. Backbone is a model downstream module that is stacked by multiple MBConv for image feature extraction. Among them, the structure of MBConv is similar to the residual block, and effective features are extracted from the input through three steps. In the first step, MBConv uses 1×1 convolution to increase the dimension of the input. The second step is to extract the deep semantic features of the feature map with increased dimension by using depthwise separable convolution (Chollet, 2017). The third step is to integrate the input of MBConv with the deep semantic features generated in the second step as the final output.

**FIGURE 3:**
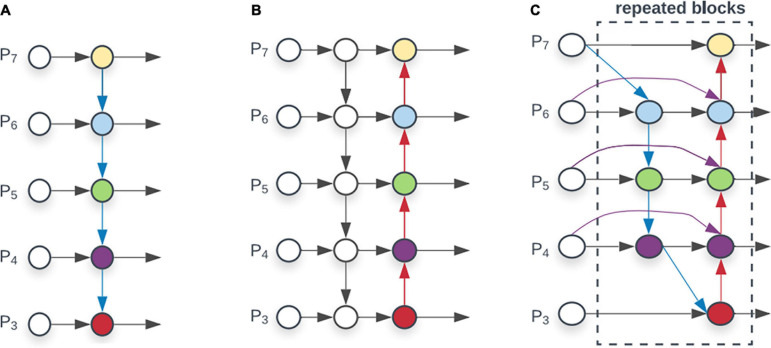
Comparison of different feature fusion layer **(A)** FPN introduces a top-down pathway to fuse multi-scale features from level 3 to7 (P3-P7). **(B)** PANet adds bottom-up pathway on top of FPN. **(C)** BiFPN with better and efficiency trade-offs.

A weighted feature fusion module BiFPN is proposed in the EfficientDet series model, shown in Figure 3. Compared with other superficial feature fusion layers such as FPN (Lin et al., 2017) and PANet (Wang et al., 2019), the weighted connection method is adopted inside BiFPN. All previous methods treat all input features equally, but different input features at different resolutions usually contribute unequally to the output features. Through 3×3 convolution and 1×1 convolution to achieve weighting of feature maps, the network model can learn the importance of different feature layers. This method makes multi-scale feature fusion more efficient. In CNN, low-level features contain more location and detailed information, but because less convolution layers are passed, they have less semantic information and more noise. The high-level features are full of semantic information, but the perception of details is poor. BiFPN combines the two features, making the feature map have the advantages of high-level feature maps and low-level feature maps. In the authentic wheat ear detection task, BiFPN enables the model to extract features at different scales. This significantly improves the model’s multi-scale detection capabilities and detection capabilities in complex backgrounds.

After BiFPN has processed the feature map of wheat ears, each pixel of the feature map will be placed anchors. In EfficientDet-D0, the number of is usually set to 9, and these have different scales and aspect ratios. Then the classification layer model judges whether each anchor point contains background or wheat ears and returns the confidence. If the confidence is higher than 0.5, the regression layer will fine-tune the upper-left and lower-right coordinates of the anchor to make it closer to the real bounding box. The result at this time cannot be directly applied to the wheat ear counting. Since the detection process is based on an anchoring mechanism, the position of the same wheat ear usually corresponds to multiple overlapping candidate boxes. The NMS algorithm is to delete those duplicate candidate boxes. If the high-confidence candidate box is overlapped by some of the low-confidence, the low-confidence candidate boxes will be deleted. After NMS, the wheat ears will be independently labeled, and the number of detection results can be counted by the computer to complete the end-to-end wheat ear counting.

### Backbone Training Using Transfer Learning

The predictive ability of the CNN model largely depends on the size of the data set. The more abundant the data, the better the CNN model’s ability to extract image features. However, not every computer vision problem can obtain sufficient data. In this case, it is extremely difficult to train a model from scratch. Transfer learning provides a simpler and faster method. Before starting to train, the backbone of the CNN model is pre-trained on a huge data set. ImageNet (Shorten and Khoshgoftaar, 2019) is a commonly used transfer learning data set. It includes more than 14 million common images, which can provide sufficient materials for CNN training. The pre-trained backbone is sensitive to the features of the image. The trained backbone is then transferred back to the model and all parts of the model are fine-tuned using experimental data. In this way, an excellent CNN model is trained with a small amount of data.

**FIGURE 4:**
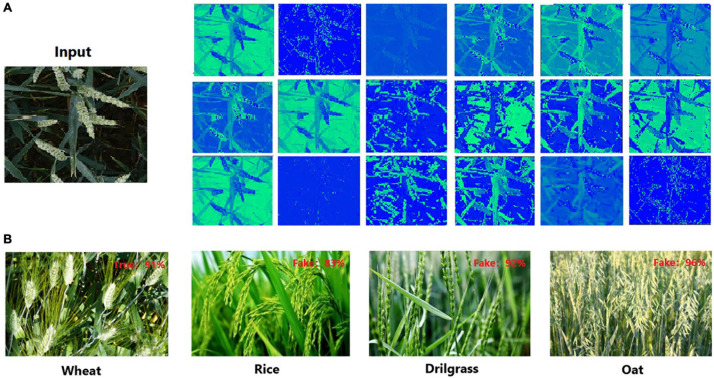
Backbone pre-training results. **(A)** The first 24 features of the fifth convolution layers for backbone. **(B)** The classification results for backbone with full connection layer and classification layer.

However, there are domain gaps in the marginal distribution of ImageNet datasets and wheat datasets, and the task similarity is weak. Due to these differences, the backbone network pre-trained on the ImageNet dataset does not have a strong perception of the wheat ear features. Such a direct transfer learning method cannot get the best backbone in wheat ear detection. A serious domain shift cannot exist between learning data and training data, so a dataset was specially constructed for the pre-training backbone in this research. For a better description, this data set is defined as D1, and the wheat ear data is defined as D0. Data set D1 consists of two parts, D0 and non-wheat data. Non-wheat data includes 2,256 rice images, 561 oat images, and 274 drilgrass images. The appearance of these three crops is very close to wheat. The D1 dataset is used to train the classification task of the EffcientDet-D0 backbone with fully connected (FC) layer and classification layer (Figure 2). The goal of classification is to distinguish whether the image is wheat. It is not easy to accurately classify these crops, not only does the backbone need to be sensitive to simple features, but it also needs to have a strong perception of high-level semantic features of wheat ears. Figure 4 shows the output of the middle layer of the backbone and the classification results.

### Wheat Ear Counting Under Occlusion Condition

Occlusion and overlap are the primary problems faced in wheat ear detection and counting. To improve the detection accuracy, these problems must be considered in the algorithm design. This article proposes an effective solution to solve the occlusion and overlap in wheat ear detection. First, in the image preprocessing stage, Random-Cutout is used to augment the image so that the model can fully learn these tricky occlusion areas. Secondly, in the model, the adoption of the CBAM attention module can refine the features of occluded wheat ears; therefore, it makes the model detect the wheat ears from the cluttered background, while reducing the interference of background and occlusion areas.

#### Random-Cutout for Occlusion Image Augmentation

**FIGURE 5:**
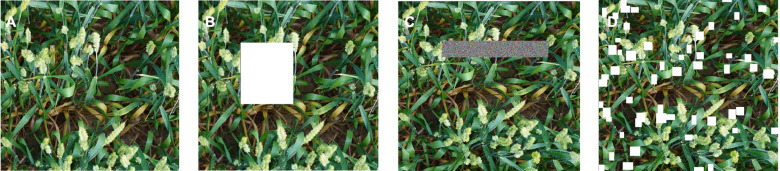
Erasing illustration for different methods: **(A)** input image, **(B)** cutout (erased rectangles marked in white), **(C)** random Erasing (erased rectangles marked with random noise), and **(D)** Random-Cutout (erased rectangles marked in white).

To broaden the diversity of samples and increase the model’s priori knowledge of the occlusion problem, an image augmentation method is proposed for dense object detection. In an image of wheat ears, the occurrence of occlusion and overlap is often related to the distribution of wheat ears. In order to simulate the occlusion under real conditions better, some rectangles are randomly erased. In the existing approaches, such as Cutout (Devries and Taylor, 2017) and Random Erasing (Zhong et al., 2020), the completely random positions of a fixed size of the image were occluded. If these methods are applied to wheat ear counting, a few wheat ears in the image may be totally occluded and these areas will be processed as noise data (Figure 5).

Random-Cutout generates occlusion area randomly to meet the wheat ear growth distribution and avoid the negative effects of excessive and insufficient occlusion on model training. Depending on the distribution of real occlusion in the images, the proposed Random-Cutout algorithm combines position and size information to generate the simulated occlusion area. In terms of the location, the probability of occlusion in dense areas of wheat ears is much higher than that in sparse areas. However, it is important to emphasize that this does not mean that occlusion does not occur in sparse areas. Occlusion and overlap are also commonly associated with wheat leaves and stems. In terms of size of random occlusion, the core is to occlude wheat ears effectively without completely losing the context information. In the wheat ear dataset, the wheat ear scales in images with different field of vision are greatly different, which means that the occlusion size generated by the algorithm cannot be set to a fixed value. When the occlusion size of a large wheat ear is applied to the images of small scales wheat ears, a lot of valid context information in the image will be erased directly. Therefore, the occlusion size generated by the Random-Cutout should be adjusted adaptively according to the size of the wheat ears in the current image.

**FIGURE 6:**
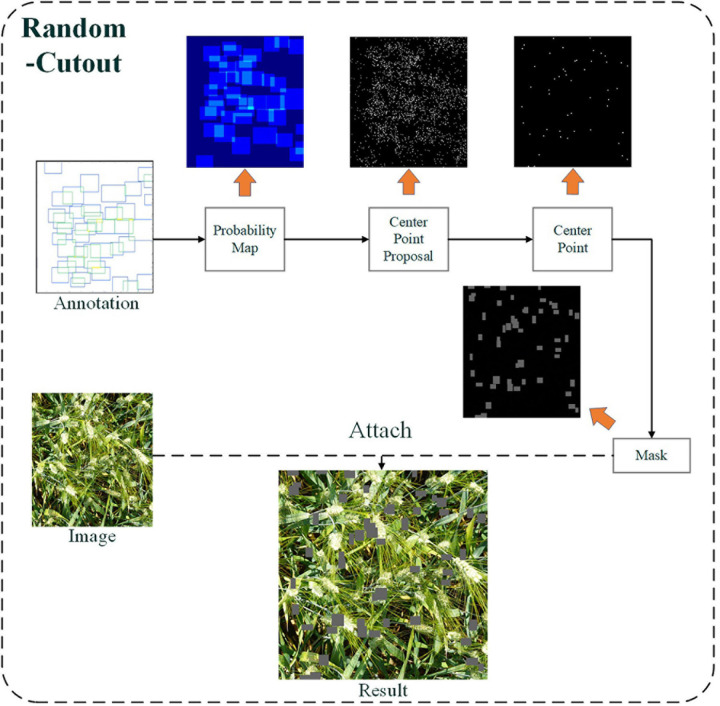
The schematic layout of the Random-Cutout.

The flowchart of the Random-Cutout is shown in Figure 6. First, Probability Map is generated according to the distribution of wheat ears in the images to determine the approximate location of the simulated occlusion. The value of each pixel is defined as a probability value *I*, in which the value of the cold color area is low, and the value of the warm color is high. Next, Center Point Proposal is generated according to the Probability Map. At this time, there may be hundreds or thousands of candidate center points, and the total number of them needs to be adjusted to a suitable value *N*. It is necessary to randomly select *N* center points from all Center Point Proposal according to the number of objects in the images. Finally, a rectangle of random length *H* and width *W* is initialized from these center points and superimposed to the original images. *H* and *W* are closely linked to the size of wheat ears in the image. We conducted a lot of experiments to determine the settings of the above parameters, which are shown in Table 1.

**TABLE 1:** The setting of hyper-parameters in Random-Cutout in this research.

	Definition of parameters	Mathematical definition
*I*	The initial probability value of each pixel point is 0.001. When wheat ears exist at this pixel point, the probability value of the pixel point is the initial value plus the number of wheat ears multiplied by 0.003	*I*_*i*_ = 0.0010.003×*n*_*i*_
*N*	1/4 of the number of wheat ears in the image	*N* = *N*_*total*_/4
*H*	Random number between one quarter of the minimum length and one quarter of the maximum length of the wheat ear in the image	$\forall H\in ⋃\left(\frac{1}{4}\times H_{m\,i\,n},\frac{1}{4}\times H_{m\,a\,x}\right)$
*W*	Random number between one quarter of the minimum width and one quarter of the maximum width of the wheat ears in the image	$\forall W\in ⋃\left(\frac{1}{4}\times W_{m\,i\,n},\frac{1}{4}\times W_{m\,a\,x}\right)$

#### CBAM for Refining Features of Partially Occluded Wheat Ears

Using the visual attention mechanism in multi-object detection model is an effective way to overcome occlusion and overlap problems. The attention module concentrates “Resources” on salience areas of the image and extracts global information from these fine-grained features. Therefore, the model can quickly filter out unwanted information and focus on the region of interest (Laskar and Kannala, 2017).

**FIGURE 7:**
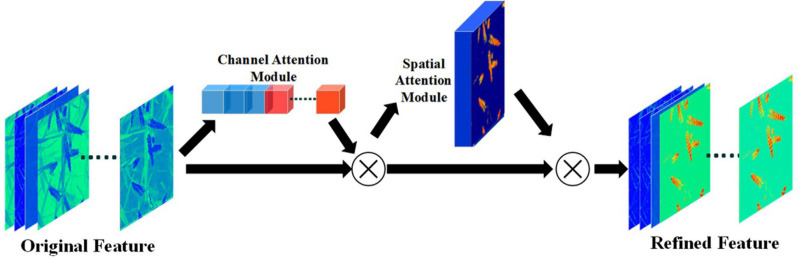
The process of CBAM module generating channel attention and spatial attention.

Convolutional block attention module (Woo et al., 2018) is one of the most effective attention modules. CBAM refines the feature map by calculating the weight of the features in space domain and channel domain (Figure 7). For feature map *F* ∈ *ℝ*^*W*×*H*×*C*^, each channel can be regarded as a feature in the images extracted by CNN. By aggregating the relations between channels in the feature map, channel attention module can obtain the “what” features that should be paid attention to in the images. Channel attention module first uses global average pooling and global max pooling operations to generate two different channel context descriptors: *F^c^*_*avg*_ and *F^c^*_*max*_, which represent average-pooled features and max-pooled features. Then these two features are input into a weight sharing module to generate a channel attention vector M_*C*_ ∈ *ℝ*^*C*×1^. The weight sharing module is a multilayer perceptron (MLP) with hidden layer. The hidden layer size is set to *ℝ*^*C*/*r*×1^, where, r is the scaling factor. After applying the shared network to each descriptor, the attention feature is generated by element-wise summation. Equation 1 shows how channel-wise attention is generated (Woo et al., 2018):

(1) $${\text{M}}_{c}\,\left(F\right)=\sigma \,\left(M\,L\,P\,\left(A\,v\,g\,P\,o\,o\,l\,\left(F\right)\right)+M\,L\,P\,\left(M\,a\,x\,P\,o\,o\,l\,\left(F\right)\right)\right)=\sigma \,\left(W_{1}\,\left(W_{0}\,\left(F_{a\,v\,g}^{c}\right)\right)+W_{1}\,\left(W_{0}\,\left(F_{m\,a\,x}^{c}\right)\right)\right)$$

where, σ represents the sigmoid function, *W*_0_ ∈ *ℝ*^*C*/*r*×*C*^,*W*_1_ ∈ *ℝ*^*C*/*r*×*C*/*r*^ indicates the weight of MLP, *W_0*, *W*_1_ share two inputs and ReLU activate function.

After generating the attention on the channel, the spatial attention can be generated through the pooling operation. Compared with channel-wise attention, spatial-wise attention is constructed more explicitly. The purpose of the spatial attention module is to obtain the prominent region in the image, that is, “where” the image needs to be paid attention to. The spatial attention module first uses max pooling and average pooling along the direction of the feature map channel to obtain two spatial descriptors: *F^s^*_*avg*_ and *F^s^*_*max*_. In order to have a larger spatial receptive field for the two descriptors, a larger pool filter is usually used in this step, e.g., 7 × 7, 15 × 15. After that, spatial attention module concatenates two spatial descriptors and uses a convolution layer to generate spatial-wise attention, Equation 2 shows how spatial-wise attention is generated (Woo et al., 2018):

(2) $${\text{M}}_{s}\,\left(F\right)=\sigma \,\left(f^{7\times 7}\,\left(\left[A\,v\,g\,P\,o\,o\,l\,\left(F\right);M\,a\,x\,P\,o\,o\,l\,\left(F\right)\right]\right)\right)=\sigma \,\left(f^{7\times 7}\,\left(\left[F_{a\,v\,g}^{s};F_{m\,a\,x}^{s}\right]\right)\right)$$

where, σ represents the sigmoid function. *f*^7×7^represents a convolution with a convolution kernel size of 7 × 7.

After generating channel-wise attention and spatial-wise attention, the feature map can be refined twice by element-wise multiplication, this process can be described as Equation 3:

(3) $$F^{\cdot }={\text{M}}_{c}\,\left(F\right)⊗FF^{\cdot \cdot }={\text{M}}_{s}\,\left(F^{\cdot }\right)⊗F^{\cdot }$$

where, *F*^⋅^ and *F*^⋅⋅^ represent the first and second refinement results of the feature map, respectively, ⊗ represent element-wise multiplication.

The wheat ears are distributed in a messy background, therefore, CBAM play an extraordinary role. In this study, five CBAM are added between the EffcientDet-D0 backbone and the feature fusion layer BiFPN (Figure 2). Five feature maps of different scales outputted by the backbone network will be used as the training input in the attention modules, so that the model can effectively get the features of different spatial information and semantic information.

### Criteria for Performance Evaluation

Evaluation indicators are objective evaluation criteria for the results of the algorithm. In different tasks, the evaluation indicators are different. In this study, counting accuracy rate (P), false detection rate (O), and frames per second (FPS) are used as performance indicators. Counting accuracy rate is the ratio between the correct number of wheat ears and the actual number of wheat ears, while false detection rate is the ratio of the number of wheat ears detected incorrectly to the total number detected. Equation 4 gives the definition of these two evaluation criteria.

(4) $$P=\frac{N_{c\,o\,r}}{N_{r\,e\,a\,l}},O=\frac{N_{e\,r\,r}}{N_{n\,u\,m}}$$

where, *N*_*cor*_ is the number of wheat ears that the model detects correctly, and *N*_*err*_ is the number of errors detected by model. *Nreal* represents the actual number of wheat ears in the test image. *Nnum* represents the total number detected by the model.

frames per second is an index to evaluate the inference speed of the model, which indicates how many images the model can process per second. Usually only when the FPS reaches 24 or more, this model is possible to achieve real-time detection. FPS is defined as shown in Equation 5:

(5) $$F\,P\,S=\frac{1}{T}$$

where, *T* denotes the time used by the model to infer the image.

### Hyper-Parameter Configuration and Learning Rate Optimization

In order to ensure reasonableness, the same hyper-parameters are set in the comparison experiments. The stochastic gradient descent (SGD) method is used to optimize the training of the loss function. Batch size and epoch are set to 12 and 300, respectively. The learning efficiency will be reduced by 50% every 30 iteration. At the same time, to prevent over-fitting, an early stopping strategy is set. When the loss of the verification dataset does not reduce or rise in 5 iterations, then the training will stop early.

Learning rate controls the speed of gradient descent during CNN training (Equation 6). If the learning rate is set too small, the convergence process of the model will be slow. If the learning rate is set too large, the gradient will oscillate repeatedly near the minimum or even fail to converge.

(6) $$\theta_{\bar{i}+1}=\theta_{i}-\alpha \,\frac{\partial }{\partial \theta_{i}}\,L\,\left(\theta_{i}\right)$$

where, θ_*i*_ represents the parameters that need to be updated during the i-th iteration, α represents the learning rate, and *L* represents the loss function.

**FIGURE 8:**
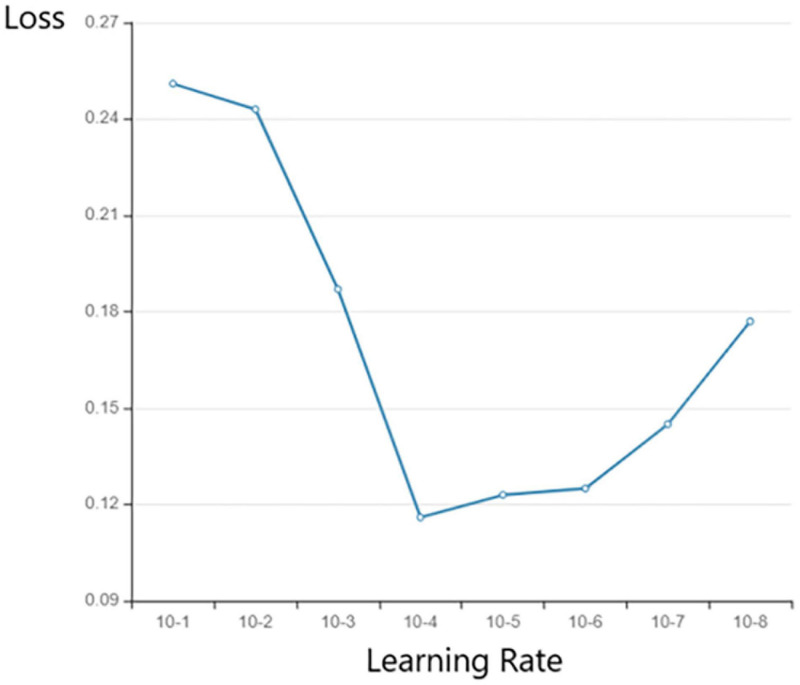
The effect of learning rate on loss.

In this study, we compared the influence of different levels of learning rate on the final loss value of the model (Figure 8) and found that the learning rate is optimal under the order of 10e-4.

It should be noted that the loss function (*L*) consists of two parts, classification loss function (*L*_*class*_) and regression loss function (*L*_*reg*_), as shown in Equation 7. The purpose of optimizing *L*_*class*_ is to allow the network to distinguish wheat ears and background, and the purpose of optimizing *L*_*reg*_ is to enable the network to locate these wheat ears accurately.

(7) $$L=L_{c\,l\,a\,s\,s}+L_{r\,e\,g}$$

## Results and Analysis

In this section, comparative experiments are first done to show that the modifications, such as transfer learning, image augmentation and CBAM, works in performance promotion. Then comparative experiments are done to show the superiority of the proposed algorithm.

### Performance Comparison With EfficientDet-D0

**FIGURE 9:**
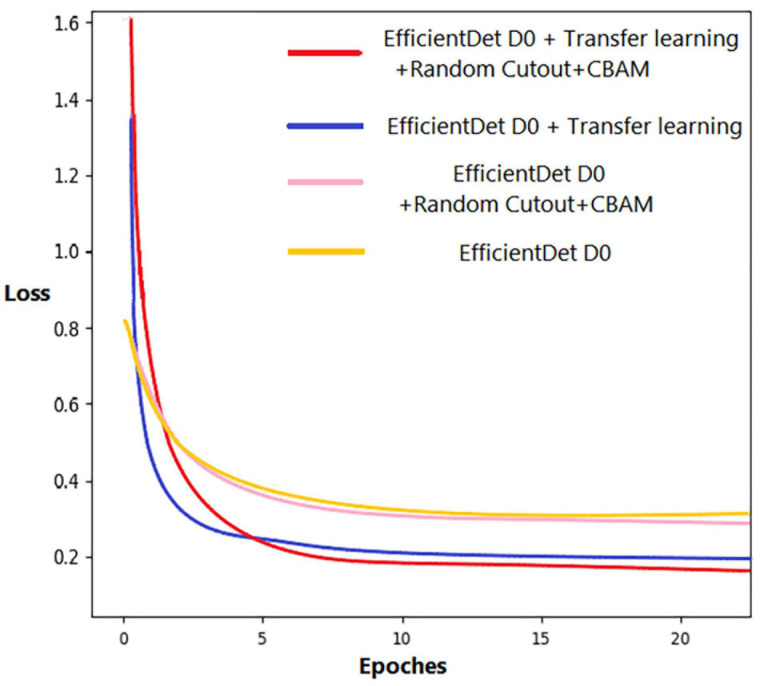
Loss function curve.

**FIGURE 10:**
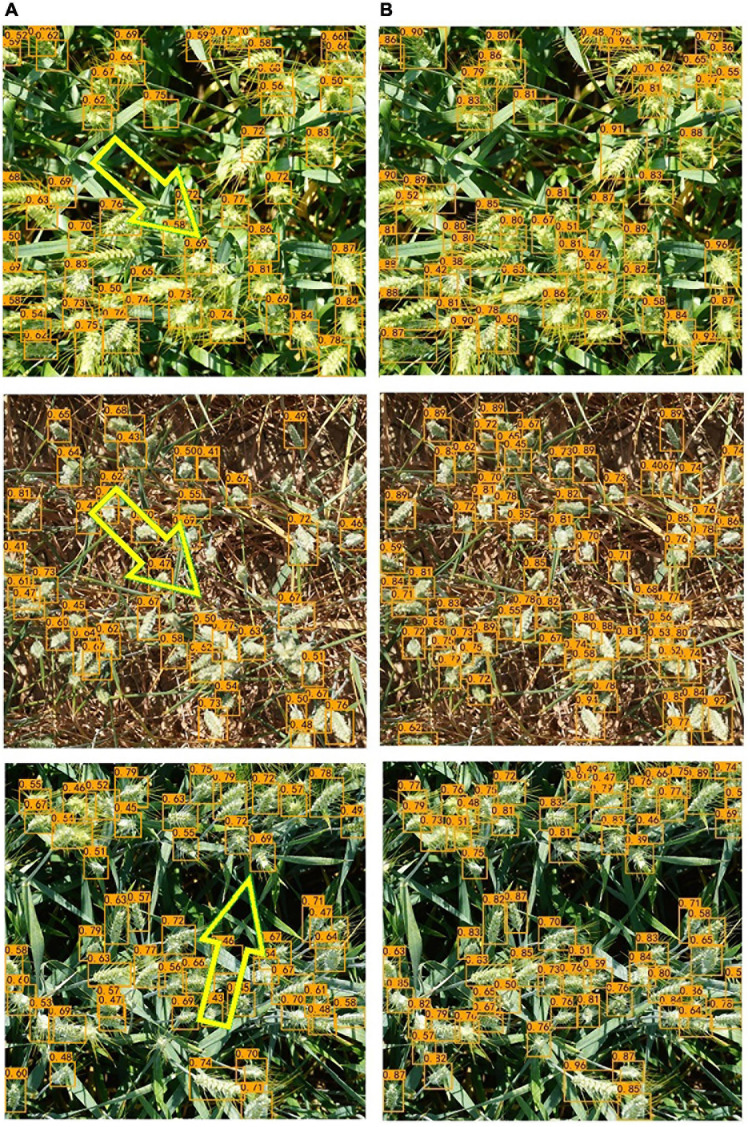
Detection results of the two networks. **(A)** EfficientDet-D0 (Some obvious missed detections are highlighted by yellow arrows). **(B)** The improved EfficientDet-D0.

In the comparison experiments, we first compared the improved EfficientDet-D0 with the original one. Figure 9 shows the loss function curve of the model in four cases during the training process. To make the difference obvious, the curves in the figure are smoothed. Regardless of whether the improved model is under transfer learning conditions, the loss value is greatly reduced. It can be seen that the transfer learning method also played a role in the experiment. The loss of the model with and without transfer learning is reduced by 0.101 and 0.122, respectively. In terms of detection ability, our improved model has been significantly improved. The detection results of EfficientDet-D0 show that there are many omissions in the intensive area, but the number of missed wheat ears with our model is significantly reduced (Figure 10).

**FIGURE 11:**
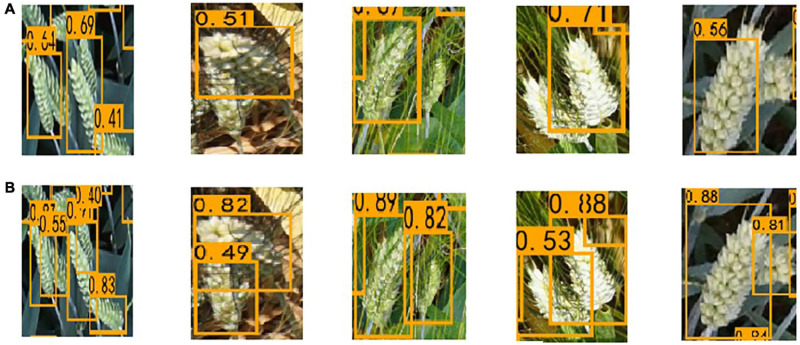
The results of the two networks on the overlap. **(A)** EfficientDet-D0. **(B)** The improved EfficientDet-D0.

At the same time, it is found that the improved EfficientDet-D0 model has dramatically reduced the impact of occlusion on the detection results. Before the improvement, the model distinguished multiple adjacent wheat ears into one, which was most serious in the dense area of wheat ears. The proposed method greatly overcomes this drawback. We selected several severely occluded images in the data set and tested them on two models, respectively; the results are shown in Figure 11. The results show whether occlusion between the wheat leaves and ears or overlap between wheat ears, the proposed network has been dramatically improved (Table 2).

**TABLE 2:** The missed detection rate of the two models under types of occlusion.

Types of occlusions	EfficientDet-D0 (%)	Proposed (%)
Overlap between wheat ears and wheat ears (265 images)	13.8	8.7
Leaves cover wheat ears (112 images)	5.2	3.3
Wheat ears were not fully photographed (81 images)	2.1	0.9

**FIGURE 12:**
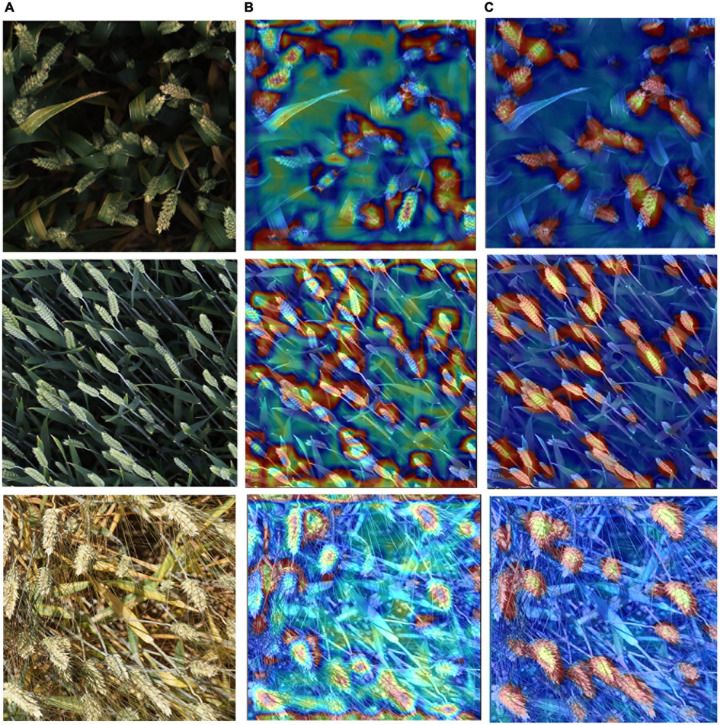
The CAM of Test images using two networks. **(A)** Test images. **(B)** The CAM of test images with EfficientDet-D0. **(C)** The CAM of test images with the improved EfficientDet-D0.

To visualize the difference of the improved EfficientDet-D0 and the original one, the Class Activation Mapping (CAM) (Zhou et al., 2016) is used to show the difference in network feature extraction (Figure 12). The thermodynamic features of different colors reveal the “attractiveness” of the regional network. Among them, the red area represents the most significant influence on the network. As the color changes from red to yellow, and finally to blue, it means that the influence gradually decreases.

### Performance Comparison of Different CNN Methods

By displaying some results in Figure 10, it can be observed that both original EfficientDet-D0 and the improved one has high ability to detect wheat ears under different lighting, background, and scales, which shows the advantages of CNN in such problems. Therefore, in order to evaluate the improved model more comprehensively, we compared it with other CNNs. In previous counting studies, models such as Faster-RCNN, YOLOV3, SSD are often used (Xu C. et al., 2020). We have compared the proposed method with these models and the results are shown in Table 3. According to the results, although the YOLOV3 and SSD models can achieve real-time detection in forwarding inference, it has a high false detection rate and a little effect on dense multi-object detection tasks. It cannot complete the task of detecting and counting wheat ears well. Faster-RCNN is a classic two-stage neural network. The counting accuracy rate of Faster-RCNN is 0.3% higher than that of Efficientdt-D0, and the false rate is 0.4% lower. But its accuracy is still about 1.3% lower than our model and its inference time is the longest.

**TABLE 3:** Peformance comparison of different CNN methods.

Method	Transfer learning	Backbone	O (Average ± STD) (%)	P (Average ± STD) (%)	FPS
YOLOv3	×	Darknet-53	7.3 ± 0.57	90.3 ± 0.46	23
SSD	×	VGG-16	8.6 ± 0.86	88.1 ± 0.14	22
Faster-RCNN	×	Resnet-50	6.3 ± 0.21	91.1 ± 0.53	16
EfficientDet-D1	×	EfficientNet-B1	6.5 ± 0.55	91.6 ± 0.45	27
EfficientDet-D0	×	EfficientNet-B0	6.7 ± 0.46	90.8 ± 0.87	**35**
Proposed	×	EfficientNet-B0	6.3 ± 0.33	92.9 ± 0.07	30
EfficientDet-D1	√	EfficientNet-B1	6.1 ± 0.14	93.1 ± 0.35	27
EfficientDet-D0	√	EfficientNet-B0	6.4 ± 0.77	92.5 ± 0.28	**35**
Proposed	√	EfficientNet-B0	**5.8 ± 0.12**	**94.2 ± 0.19**	30

*The bold font represents the best result in the experiment.*

We also did some experiments to compare the higher version of EfficientDet-D0 (i.e., EfficientDet-D1). The accuracy of the EfficientDet-D1 increased by 0.7% compared with the EfficientDate-D0 model and the improved EfficientDet-D0 increased by 1.6% (Table 3). Since EfficientDet-D1 is a general-purpose object detection model that improves accuracy by expanding the size of the backbone and feature fusion modules to extract better feature expressions, this results in a decrease in the effective inference speed of EfficientDet-D1 by 22%. In contrast, the improved EfficentDet-D0 model was designed specifically for wheat ear detection to improve accuracy by reducing occlusion interference. CBAM reduced the inference speed by about 15%, but this was the tradeoff with the improvement in accuracy. In terms of false detection rate, the improved EfficientDet-D0 is 0.3% lower than EfficientDet-D1 and 0.6% lower than EfficientDet-D0. Although the accuracy increases, the error rate of the improved model does not decrease significantly. The reason is to ensure that as many ears as possible are detected in the post-processing process, the confidence threshold is usually set to a small value, which will cause some proposed regions that do not contain ears to be leaked.

From the results in section “Performance Comparison *With* EfficientDet-D0” and section “Performance Comparison of Different CNN Methods,” it can be seen that transfer learning is an effective strategy in wheat ear detection. In transfer learning, the data do not need to be finely labeled, and only the categories they belong to are roughly labeled. After using transfer learning, the false detection rate of EfficientDet-D1, EfficientDet-D0 and the improved EfficientDet-D0 was reduced by 0.4%, 0.3% and 0.5%, while the counting accuracy rate was increased by 1.5, 1.7, and 1.3%, respectively.

## Conclusion

In this article, we proposed a novel wheat ear counting algorithm. Importantly, we focus on the occlusion and overlap problems that exist under the actual growth conditions of wheat ears. Farmers and breeders take images of wheat under a certain area in the wheat field and our proposed algorithm can automatically calculate the number of wheat ears in that area, which is helpful to evaluate and predict the level of wheat yield.

The main contributions come from the three key procedures of the proposed method. First, the transfer learning method is employed to extract the high-level semantic features of wheat ears. Secondly, an image augmentation method Random-Cutout is proposed to simulate occlusion in real wheat images. Finally, convolutional block attention module (CBAM) is adopted into the EfficientDet-D0 model to refine the features and pay more attention to the wheat ears.

Extensive experiments show that the counting accuracy of the proposed algorithm reaches 94% and false detection rate is 5.8%. The performance evaluation shows that the proposed method is invariant to illumination and scale changes. Simultaneously, the proposed method had high accuracy and strong robustness for occlusion and overlap problem. We firmly believe that human beings will benefit from automatic wheat ear counting by machines, thereby reducing manual counting errors. Moreover, it greatly reduces the labor cost. The proposed model can be used as a post-processing method to plan the wheat harvesting and storage.

The methods used in this research can achieve accurate counting of wheat ears, but the research will never stop here. In the future, we will envisage using this method in more crop counting work such as apple counting, etc. Moreover, we will apply the Random-Cutout image augmentation method to more fields, not limited to agriculture, to prove its robustness to solve the occlusion problem.

## Data Availability Statement

The original contributions presented in the study are included in the article/supplementary material, further inquiries can be directed to the corresponding author.

## Author Contributions

YW performed the experiments and analyzed the results. YQ designed and performed the experiments, and wrote the manuscript. JC wrote the manuscript. All authors contributed to the article and approved the submitted version.

## Conflict of Interest

The authors declare that the research was conducted in the absence of any commercial or financial relationships that could be construed as a potential conflict of interest.
